# Evaluation, intervention, and follow-up of patients with diabetes in a primary health care setting in Brazil: the importance of a specialized mobile consultancy

**DOI:** 10.1186/s13098-016-0173-1

**Published:** 2016-08-08

**Authors:** Wilson Eik Filho, Letícia Pastorelli Bonjorno, Ana Julia Mendes Franco, Márcia Lorena Alves dos Santos, Eniuce Menezes de Souza, Sonia Silva Marcon

**Affiliations:** 1 Endocrinology Unit, Department of Medicine, Universidade Estadual de Maringá, and Postgraduate Program in Health Sciences, Health Sciences Center, Universidade Estadual de Maringá, Maringá, Parana Brazil; 2 Medical School, Universidade Estadual de Maringá, Maringá, Parana Brazil; 3 Department of Statistics, Universidade Estadual de Maringá and Postgraduate Program in Biostatistics, Universidade Estadual de Maringá, Maringá, Parana Brazil; 4 Postgraduate Program in Health Sciences, Health Sciences Center, Universidade Estadual de Maringá, Maringá, Parana Brazil; 5 Avenida Mandacarú, 1590-Zona 07, Maringá, PR CEP: 87083-240 Brazil

**Keywords:** Type 2 diabetes mellitus, Primary health care, Health education

## Abstract

**Background:**

Studies show that educational interventions improve glycemic control in patients with diabetes mellitus (DM), reducing the occurrence of complications associated with the disease.

**Objectives:**

To evaluate the effects of a mobile DM consultancy on clinical and laboratory parameters, disease knowledge, and quality of life in patients with type 2 DM (T2DM) at a primary health care network in Brazil.

**Methods:**

Randomized clinical trial conducted in a city in southern Brazil with 52 patients with T2DM receiving care at a primary health care setting. The intervention lasted for 6 months and consisted of a follow-up with an endocrinologist (five meetings), treatment adjustment based on clinical evaluation and laboratory tests, and educational activities with conversation maps in DM. The statistical analysis included comparison and association tests, considering p values ≤0.05 as statistically significant.

**Results:**

The mean age of the patients was 63.8 years. Most participants were female (63.5 %), had low educational level (59.6 %) and family history of T2DM (71.2 %), used only oral hypoglycemic agents to manage their DM (73.2 %), presented unfavorable anthropometric and laboratory parameters, a high or medium risk of complications (84.6 %), and inadequate glycemic control (67.3 %; with 71 % of the high-risk patients presenting a HbA1c level >9 %). Adjustment in pharmacological treatment was required in 63.5 % of the patients. After the intervention, we observed a significant 0.46 % decrease in mean HbA1c level (p = 0.0218), particularly among individuals with inadequate glycemic control (0.71 %; p = 0.0136). Additionally, there was an increase in disease knowledge scores and a significant decrease in mean body mass index, waist circumference, and disease impact scores.

**Conclusion:**

The intervention improved glycemic control and disease knowledge, reduced the values of body mass index and waist circumference, and the impact of the disease on patients’ lives. This indicates that care and educational measures improve the experience of the patients with DM and control of the disease.

## Background

Diabetes mellitus (DM) is a disease with strong social impact, associated with high morbidity and mortality rates that result from long-term microvascular and macrovascular complications [1]. The prevalence of DM is increasing worldwide, despite international efforts to control the disease. Data published by the International Diabetes Federation (IDF) show an estimated 8.8 % prevalence of DM in individuals between 20 and 79 years of age (415 million individuals) and point to an estimated 642 million patients affected with the disease in 2040, a 10.4 % prevalence [2]. In Brazil, it is estimated that the number of adult patients with DM increased from 4.3 million in 2000 [3] to 14.3 million in 2015, and is expected to increase to 23.2 million in 2040. This will project the country to occupy the 4th position in number of patients with DM worldwide [2].

Two important multicenter studies, the Diabetes Control and Complications Trial (DCCT) and the United Kingdom Prospective Diabetes Study (UKPDS), have demonstrated benefits with strict blood glucose control. The DCCT [4], conducted with individuals with type 1 DM (T1DM), has shown that the maintenance of blood glucose levels close to normal values with intensive insulin therapy reduces the incidence and severity of chronic complications. The UKPDS [5], in turn, has demonstrated that improved glycemic control may reduce the risk of development of chronic complications in type 2 DM (T2DM).

In Brazil, despite the available therapeutic options, efforts of teams specialized in the treatment of DM [6], and government initiatives (such as a system of registration and follow-up of hypertensive and diabetic patients [7], instituted in primary health care), the glycemic control of diabetic patients is worse than expected. In a Brazilian multicenter study involving 6671 adults with T1DM and T2DM [8], 76 % of the patients had an inadequate glycemic control (HbA1c ≥7 %). This rate was 90 % among patients with T1DM and 73 % in those with T2DM. In another study conducted in Goiânia (Goiás) [9] including only individuals with T1DM, the rate of inadequate control was 81.7 %. Poor glycemic control is also an international problem. A study conducted in the U.S., for example, has shown that approximately 50 % of the individuals with DM in that country have poor glycemic control [10].

Education in DM increases patients’ self-care, improving their glycemic control and knowledge of the disease [6]. Evidence suggests that educational programs conducted in groups improve cost-effectiveness, particularly in adults with T2DM [11, 12]. In patients with T1DM, the improvement is mainly observed in the psychosocial sphere [13]. In Brazil, a randomized, prospective, multicenter study including individuals with T1DM and T2DM, known as *Projeto DOCE*^*®*^ (Diabetes Aiming Control and Education, *Diabetes Objetivando Controle e Educação*), developed by a multiprofessional team, showed significant improvement in blood glucose control, but no variation in quality of life scores [14–16].

Considering the imbalanced distribution of endocrinologists in Brazil, where most specialists (76.08 %) are concentrated in the country’s southeastern and southern areas [17], the current challenge is to provide better job opportunities and professional training continuity in less served regions, allowing a more balanced distribution of specialists operating both in public and private health sectors, and in small and large urban centers.

Considering the precariousness of the glycemic control in patients with DM worldwide, and the direct implications that poor glycemic control imposes on the risk of development of complications and health expenses, the aim of this study was to evaluate the effects of the action of a mobile consultancy unit specialized in DM on clinical and laboratory parameters, as well as on the degree of knowledge of and satisfaction with the disease in patients with T2DM in a primary health care network in Brazil.

## Methods

### Delineation and population

**Fig. 1:**
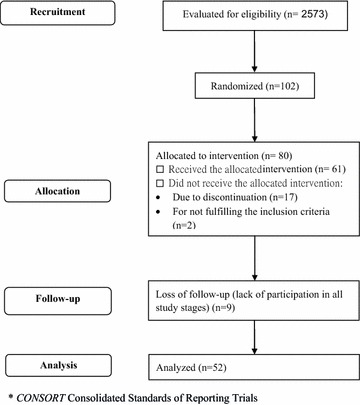
Study flow diagram

This is a randomized, non-placebo-controlled clinical trial (Fig. 1) with individuals with T2DM, developed in a primary care setting in the city of Maringá (Paraná) [18], southern Brazil, between October, 2014, and August, 2015. The city has 30 primary health units (*Unidades Básicas de Saúde,* UBS) and the study was conducted in nine of these units. To select the UBS for inclusion in the study, we took into account the division of the city into three homogeneous areas according to the economical resources and demographic structure of the population [19]: G1 (more favorable, six UBS), G2 (intermediate, four UBS), and G3 (less favorable, 20 UBS). The number of UBS and patients per UBS was proportional to the number of individuals with DM registered in each area (two UBS in group G1, two in group G2, and five in group G3). The total number of eligible individuals in all nine UBS was 2573.

We adopted the following inclusion criteria: diagnosis of T2DM, age between 18 and 80 years, and interest in and availability to participate in the activities of the study. The patients were selected randomly and proportionately to the population of individuals with T2DM in each of the nine UBS. The participation was entirely voluntary, and the selected individuals could be replaced by the next individual on the list for a maximum of three times. We excluded individuals who presented important mental disorder, severe associated diseases, substantial decrease in visual acuity, and impaired ambulation. Of 80 contacted patients, 61 agreed to participate in the study and 52 participated in all stages of the study.

### Data collection

The data were collected at each UBS at three moments: initial visit (V1; before the intervention, during the selection of the individual for the study), intermediate visit (V4; 3 months after the first intervention), and final visit (V5; 6 months later). In all meetings, we consulted the electronic records of the patients to analyze the data related to their clinical condition and treatment regimen, and verify their anthropometric data and serum HbA1c results. Levels of serum HbA1c were determined with an Ames analyzer (Bayer Diagnostics, DCA 2000). The normal range for HbA1c values determined with this analyzer is between 4.2 and 6.5 % (mean 5.0 ± 0.35 %, 95 % confidence interval 4.3–5.7 %) [20, 21]. The correlation coefficient of HbA1c values obtained with the Ames analyzer compared with values obtained by high-performance liquid chromatography (HPLC) is 0.95 [22]. The other laboratory tests and the questionnaires were only performed/applied at V1 and V5.

To collect the data, we applied the following three questionnaires

(1) Identification questionnaire: sociodemographic characteristics and lifestyle habits (sex, age, skin color, educational level, family income, smoking, alcohol abuse, and practice of physical activity), anthropometric data (weight [kg], height [m], BMI [kg/m^2^], waist circumference [WC; cm], hip circumference [HC; cm], WC/HC ratio, and blood pressure [BP; mmHg]), clinical data (type of treatment, disease duration, family history, comorbidities, metabolic control, and degree of risk of complications), and laboratory data (complete blood count, fasting blood glucose, HbA1c, creatinine, potassium, lipid profile, alanine aminotransferase [ALT] and aspartate aminotransferase [AST]), TSH, urinalysis, and microalbuminuria. All laboratory tests were performed by the Health Department of Maringá (Paraná), and only those tests performed up to 6 months before the beginning of the study were included in the analysis.

We considered as smokers those individuals who were current smokers, independent of the number of cigarettes they smoked. Alcohol abuse was defined as alcohol ingestion on a single occasion of 60 g or more of in the previous 30 days [23]. We considered as sedentary those individuals who presented a caloric expenditure ≤1.5 kcal/kg/h [24]. The reference values adopted for the anthropometric parameters and risk factors were: BMI: <18.5, low weight; 18.5–24.9, normal weight; 25–29.9, overweight; 30–34.9, grade I obesity, 35–39.9, grade II obesity; ≥40, grade III obesity; WC: men: ≤94, normal and ≥102, increased; women: ≤80, normal and ≥88, increased; WC/HC: men: ≤0.9, normal; women: ≤0.85, normal; abnormal BP: ≥140/90 [25, 26]. The metabolic control was classified as good (HbA1c ≤7 %), regular (HbA1c >7 % and ≤9 %), or poor (HbA1c >9 %) [27].

We also considered individuals with HbA1c level >7 % (which includes those with regular and poor control) as having inadequate glycemic control. The risk of complications was graded based on criteria from the program Qualification of Primary Health Care (*Qualificação da Atenção Primária à Saúde*, APSUS), adopted by primary care professionals in the state of Paraná [28]. Regarding the DM [29], the patients were classified into three levels: low risk (fasting blood glucose or glucose intolerance determined in the oral glucose overload test), medium risk (DM and adequate metabolic and BP controls, without hospitalizations due to acute complications in the past 12 months, without chronic complications), high risk (DM and inadequate metabolic and BP control, or adequate metabolic and BP controls, but with hospitalizations due to acute complications in the past 12 months and/or presence of chronic complications).

(2) Questionnaire assessing DM knowledge: we applied the DKN-A (Diabetes Knowledge Scale Questionnaire), validated for the Brazilian population [30]. This questionnaire comprises 15 items related to DM knowledge (basic physiology, food groups and substitutions, management of DM in intercurrent events, and general principles of DM care) [30]. The responses are presented in a multiple-choice scale and the total score ranges from 0 to 15 points. Scores ≤7 indicate unsatisfactory knowledge, and scores ≥8 reflect satisfactory knowledge [31].

(3) Diabetes Quality of Life Measure-Brazil (DQOL-BR), validated for the Brazilian population [32]: this questionnaire comprises 44 questions distributed in four domains, namely: satisfaction (15 questions), impact (18 questions), social/vocational concerns (seven questions), and concerns related to the DM (four questions). Answers are given on a Likert-type scale of five points. In the overall score or in the score for each domain, the closer the result is to 1, the greater the satisfaction with the quality of life related to the disease.

### Intervention

The intervention was carried out exclusively by the principal investigator, and occurred in three phases that included (1) a follow-up by an endocrinologist with adjustment of pharmacological and non-pharmacological treatments based on clinical and laboratory evaluation, and (2) educational activities with the application of conversation maps (CMs) in DM. The CMs were developed by the American health education company Healthy Interactions in partnership with a pharmaceutical company [33]. The maps feature an interactive content and are presented by a facilitator trained by a specialized instructor. In all, there are seven 91.4 × 138.6 cm maps that present varying aspects of the disease, such as etiopathogenesis, pathophysiology, diet, physical activity, types of treatment and monitoring, as well as control and complications associated with the disease. In the present study, we used three CMs (numbers 1 and 2), entitled, respectively: “How the body and diabetes work” and “Healthy diet and physical activity” which were presented at two meetings held in the same week—visits 2 (V2) and 3 (V3). CM number 3 (“Drug treatment and blood glucose monitoring”) was applied 3 months later, at the fourth visit (V4). The sessions with CMs lasted two to 3 h and occurred at the UBS in groups of 5–10 individuals in the meetings V2, V3 and V4, which had a total duration of approximately 4 h.

The adjustment of the non-pharmacological and pharmacological therapies was performed according to the guidelines of the Brazilian Diabetes Society (*Sociedade Brasileira de Diabetes*) [6], starting preferably at initial meeting (V1) before sessions with the conversation maps, but were made occasional treatment adjustments mainly in V4 and V5, due to the availability of additional schedules to individualize all the proposed activities.

The endocrinologist in charge of the study was authorized by the Municipal Health Department and consented by the UBS doctors to manipulate the medical records, adjust treatment, and request laboratory tests when necessary. In the last visit (V5), the patients were encouraged to maintain the recommendations proposed in the study and continue to adhere to the UBS educational programs. The health professionals at the UBS were informed about the results obtained in the study and the availability of the endocrinologist for future contacts.

### Statistical analysis

Quantitative variables were assessed for normality with the Shapiro–Wilk test. Descriptive variables with a significance level of 5 % in the test are presented as mean and standard deviation. Variables without normal distribution are presented as median and quartiles range (percentiles 25 and 75 ‰). To compare mean and median values between V1 and V5, we used paired Student’s *t* test and Wilcoxon test, respectively, according to the distribution of each variable, considering a significance level of 0.05. To evaluate the homogeneity and similarity of all three groups, we used Chi square, Fisher’s exact, and Kruskal–Wallis tests, according to each variable analyzed. All analyses were performed with the software R (The R Foundation for Statistical Computing, Vienna, Austria).

### Ethics

The study was conducted according to national and international ethics standards for research involving human subjects (protocol CAAE: 25897314.7.0000.0104).

## Results

### Sociodemographic and clinical data

Table 1 shows the general characteristics of the study participants according to assignment group, consumer class, and demographic structure. We observed no significant differences in general characteristics among the groups, except for family history (DM), and WC/HC values in women, thus enabling the groups to be analyzed together. As shown in Table 1, of the 52 patients included in the study, most (63.5 %) were female, white (80.8 %), and had a low educational level (59.6 %). The mean age was 63.8 years (38–80 years), and the most frequent age group was that of individuals aged 60 years or more (69.2 %). The monthly family income of most participants (90.5 %) varied between two and five minimum wages (approximately 500–1500 US Dollars).

**Table 1:** General characteristics of the patients at the beginning of the study (V1)

	Variables	Group 1	Group 2	Group 3	Total	p value
Gender	Female	9 (60.0)	12 (85.7)	12 (52.2)	33 (63.5)	0.110*
	Male	6 (40.0)	2 (14.3)	11 (47.8)	19 (36.5)	
Age	<60 years	2 (13.3)	3 (21.4)	11 (47.8)	16 (30.8)	0.068**
	≥60 years	13 (86.7)	11 (78.6)	12 (52.2)	36 (69.2)	
Race	White	14 (93.3)	10 (71.5)	18 (78.3)	42 (80.8)	0.326**
	Non white	1 (6.7)	4 (28.6)	5 (21.7)	10 (19.2)	
Educational level	≤8 years	9 (60.0)	11 (78.6)	13 (56.5)	33 (63.4)	0.380*
	>8 years	6 (40.0)	3 (21.4)	10 (43.5)	19 (36.6)	
Smoking	No	14 (93.3)	14 (100.0)	23 (100)	51 (98.1)	0.557**
	Yes	1 (6.7)	0	0	1 (1.9)	
Alcoholism	No	13 (86.7)	14 (100.0)	23 (100)	50 (96.2)	0.148**
	Yes	2 (13.3)	0	0	2 (3.8)	
Physical Activity	Mild/moderate	7 (46.7)	7 (50.0)	10 (43.5)	34 (46.1)	0.927*
	Sedentary	8 (53.3)	7 (50.0)	13 (56.5)	28 (53.9)	
Treatment	NP	0	0	2 (8.7 %)	2 (3.8 %)	0.179**
	OM	9 (60 %)	12 (85.7 %)	17 (74 %)	38 (73.2 %)	
	OM/I	6 (40.0 %)	2 (14.3 %)	4 (17.3 %)	12 (23.0 %)	
DM duration (years)		10 (8, 23.5)	11 (3.5, 19.3)	6 (3, 11)	9 (4, 15)	0.083***
BMI		30.37 ± 6.2	30.32 ± 4.5	29.67 ± 3.9	30.05 ± 4.7	0.470***
WC	Female	102.7 ± 14.6	98.4 ± 9.6	104.62 ± 10.3	101.8 ± 11.3	0.356***
	Male	109.4 ± 12.2	117 ± 12.0	103.1 ± 10.0	106.6 ± 11.3	0.320***
WC/HC	Female	0.94 ± 0.03	0.93 ± 0.03	0.99 ± 0.03	0.95 ± 0.04	0.001***
	Male	0.99 ± 0.04	1.04 ± 0.04	0.99 ± 0.05	0.99 ± 0.05	0.358***
Family	No	8 (53.3)	4 (28.6)	3 (13.0)	15 (28.8)	0.027**
History (DM)	Yes	7 (46.7)	10 (71.4)	20 (87.0)	37 (71.2)	
Complication	Low	2 (13.3)	3 (21.4)	3 (13.0)	8 (15.4)	
Risk	Medium	3 (20.0)	6 (42.9)	11 (47.9)	20 (38.4)	0.371**
	High	10 (66.7)	5 (35.7)	9 (39.1)	24 (46.2)	

*NP* non-pharmacological treatment; *OM* oral medications;* OM/I* oral medications and insulin; *BMI* body mass index; *WC* waist circumference; *WC/HC* waist circumference/hip circumference ratio

* Chi square test

** Fisher exact test

*** Kruskal–Wallis test

As for lifestyle habits (Table 1) only one individual was a smoker, most participants (96.2 %) did not abuse alcohol, and less than half of the individuals (46.1 %) practiced mild or moderate physical activity.

Regarding clinical variables, we observed that the median disease duration was 9 years, ranging from 1–50 years. Most patients had regular or poor blood glucose control (67.3 %), were classified as having a high or medium risk of complication (84.6 %), reported having family history of T2DM (71.2 %), and were only treated with oral antidiabetic agents (73.2 %), mainly metformin at a dose between 500 and 2550 mg/day, sulphonylureas (glibenclamide 5–20 mg/day or gliclazide MR 30–120 mg/day). Three patients (5.8 %) used vildagliptin 100 mg/day, and one patient used pioglitazone 30 mg/day.

The most frequent diseases associated with DM were hypertension (75 %), obesity (46.1 %), dyslipidemia (36.5 %), and hypothyroidism (13.46 %). It is noteworthy that of the 39 patients with associated hypertension at the initial evaluation, 15.4 % had a BP level ≥140/90 mmHg (data not shown).

As for the anthropometric indices, the mean BMI, WC, and the WC/HC ratio in both genders showed evidence of an association between overweight and DM.

The results of laboratory tests showed anemia in four patients (7.7 %) and absence of significant changes in leukocyte count, platelets, ALT, AST, potassium, lipid profile, TSH, and urinalysis. The combined analysis of creatinine and microalbuminuria showed the occurrence of diabetic nephropathy in 17.3 % of the patients.

The mean serum HbA1c level was 8.91 %. The glycemic control was good in 17 patients (32.7 %), regular in 16 patients (30.8 %), and poor in 19 patients (36.5 %). Thus, 35 patients (67.3 %) presented inadequate glycemic control. The median serum HbA1c level was 7.75 % (6.95–10.1 %). A total of 50 % of the individuals presented an HbA1c level between 7.0 and 10.1, and 25 % above 10.1 %.

### Knowledge about DM and quality of life related to the disease

In relation to the knowledge about DM, we observed that the median score was 8.5, and only a little more than half of the patients (51.9 %) achieved a score ≥8. As for satisfaction with life related to the disease, we found that overall and for each of the domains, the scores were near 2, with the exception of the domain social/vocational concerns, in which the score was close to 1.

### Evaluation of the intervention

Clinical and laboratory evaluation pointed out to a requirement for adjustment in pharmacological treatment in 33 patients (63.5 %). In eight of these patients, the insulin dose was adjusted, in five cases insulin was started, and in 28 patients, either the insulin dose was adjusted, or the oral antidiabetic class was modified alone or in combination with the insulin adjustment. It is noteworthy that in most cases we maintained those medications available without cost in the public health network. Only four individuals were able to afford other classes of oral antidiabetics not freely available (pioglitazone 30 mg/day, linagliptin 5 mg/day, and dapagliflozin 10 mg/day).

**Fig. 2:**
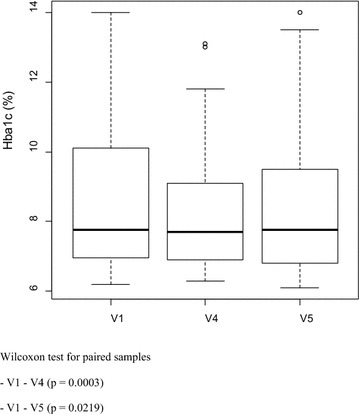
Box plot of HbA1c levels before and after the intervention

**Fig. 3:**
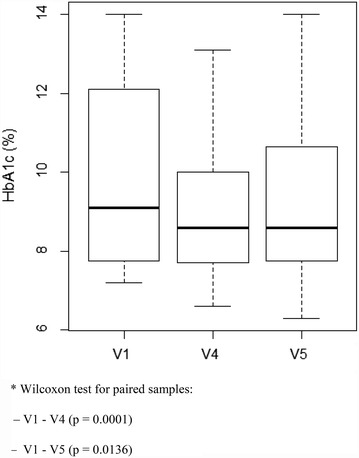
Box plot of HbA1c levels before and after the intervention in patients with inadequate glycemic control

We observed a mean reduction of 0.69 % in HbA1c levels between V1 and V4 (p = 0.0003) and 0.46 % between V1 and V5 (p = 0.0219) (Fig. 2). When we considered only those patients with inadequate glycemic control (67.3 %), the improvement in HbA1c was more substantial (Fig. 3), both from V1 to V4 (−1.07 %, p = 0.0001), as well as from V1 to V5 (−0.71 %, p = 0.0136).

The mean HbA1c level in individuals who required adjustments in their pharmacological treatment decreased from 9.61 to 9.01 %, a reduction of 0.6 % in 6 months of follow-up (p = 0.445). In the group of patients without requirement of pharmacological adjustment (36.5 %, n = 19), the mean HbA1c level decreased from 7.69 to 7.5 %, a reduction of 0.19 % (p = 0.9883).

In Table 2, we present a comparison of the initial and final values of the anthropometric variables and some laboratory tests according to the glycemic control. There was a statistically significant BMI reduction in individuals with good glycemic control at the beginning of the study, and a reduction in WC values in those with good and regular glycemic control at baseline.

**Table 2:** Variation of anthropometric and laboratory data during the study

Variables	Glycemic control (HbA1c)	V1	V5	p value
	Good	29 (28.2, 31.1)	28.9 (26.8, 30.3)	0.0157*
BMI	Regular	31.1 (27.9, 33.5)	30.5 (28.1, 33.5)	0.1031*
	Poor	28.1 (25.5, 32.4)	28.8 (25.5, 32.8)	0.8788*
	Total	29.4 (27.5, 32.6)	29.05 (26.5, 32.7)	0.0296*
	Good	103.58 ± 10.02	101.14 ± 10.06	0.0103**
AC	Regular	105.06 ± 11.53	103.09 ± 10.60	0.0316**
	Poor	102.28 ± 12.92	101.81 ± 11.46	0.5240**
	Total	103.56 ± 11.44	101.99 ± 10.57	0.0014**
	Good	0.98 ± 0.04	0.97 ± 0.04	0.6522**
AC/hip	Regular	0.94 ± 0.04	0.94 ± 0.03	0.9280**
	Poor	0.98 ± 0.05	0.98 ± 0.03	0.7626**
	Total	0.97 ± 0.05	0.96 ± 0.04	0.6155**
	Good	98 (91.5, 121.5)	111 (104, 125.5)	0.4558*
Fasting glycemia	Regular	137.5 (124, 154)	128 (114.5, 168)	0.3453*
	Poor	214 (136.5, 221.5)	180 (143, 200.5)	0.3486*
	Total	135 (107.5, 160)	133 (112, 179.2)	0.4356*
	Good	185.35 ± 26.80	184.26 ± 40.16	0.9662**
Total cholesterol	Regular	172.71 ± 36.43	173.93 ± 37.93	0.5583**
	Poor	173.27 ± 43.05	170.68 ± 42.84	0.5230**
	Total	177.33 ± 32.84	175.83 ± 40.15	0.9454**
	Good	44.5 (38, 62.5)	43 (39, 55)	0.2892*
HDL cholesterol	Regular	38.5 (36, 43.5)	40 (36, 46.5)	0.7892*
	Poor	37 (33.5, 44)	38 (34, 44.5)	0.9645*
	Total	39 (36, 45.5)	40 (35, 47)	0.3917*
	Good	115.17 ± 34.30	108.77 ± 38.57	0.4871**
LDL cholesterol	Regular	105.38 ± 31.66	101.37 ± 32.21	0.9848**
	Poor	99.48 ± 33.25	94.57 ± 33.97	0.5291**
	Total	107.23 ± 32.84	101.13 ± 34.71	0.4285**
	Good	24.5 (19.5, 28.5)	26.6 (19.2, 31.7)	0.7354*
VLDL cholesterol	Regular	24.5 (21, 31.5)	24 (21.6, 36.1)	0.0546*
	Poor	33 (22.1, 48)	32.5 (21.6, 42.25)	0.9658*
	Total	26 (20, 32.5)	28.2 (20.9, 37.25)	0.3790*
	Good	123.5 (98.25, 145)	133 (96,158.5)	0.7354*
Triglycerides	Regular	124.5 (107, 161.5)	120 (108,180.5)	0.0691*
	Poor	167 (112.5, 241)	170 (110, 226)	0.9658*
	Total	131 (101, 165.5)	145 (106, 187)	0.4152*
	Good	1 (0.7, 1.15)	0.85 (0.7, 1.12)	0.9999*
Creatinine	Regular	0.9 (0.75, 0.98)	0.8 (0.75, 0.9)	0.9643*
	Poor	0.9 (0.81, 0.97)	0.8 (0.6, 1.17)	0.7193*
	Total	0.9 (0.7, 1)	0.8 (0.7, 1.1)	0.7567*
	Good	12.2 (5.6, 45.7)	7.8 (5.6, 22.1)	0.4498*
Micro albuminuria	Regular	8.4 (3.6, 20.5)	7.5 (5.0, 21.3)	0.4131*
	Poor	9.9 (5.2, 17.2)	7.6 (5.5, 32.7)	0.4887*
	Total	10.35 (4.8, 25.6)	7.6 (5.4, 28.5)	0.7229*

*BMI* body mass index; *WC* waist circumference; *WC/HC* waist circumference/hip circumference ratio

* Wilcoxon matched pairs test

** Paired *t* test

As for the assessment of BP, the percentage of hypertensive individuals who presented a BP ≥140/90 mmHg decreased from 15.4 to 5.8 %.

Regarding the subjective evaluation of knowledge about the disease and its impact on the lives of individuals with T2DM, Table 3 shows a comparison of the results obtained with the application of the questionnaires DKN-A and DQOL-BR between V1 and V5, according to the quality of the glycemic control in the beginning of the study. Considering the knowledge about the disease, the median score increased from 8.5 (5.5, 10.5) to 9.0 (7.00, 11.0) and the proportion of individuals who achieved a score greater than or equal to 8 increased from 51.9 to 65.4 %. We observed a significant improvement in disease knowledge level among individuals who at the beginning of the study had good glycemic control. As for the DQOL-BR questionnaire, we observed a significant reduction (p < 0.05) in the total scores and in the impact domain scores in the group of patients with poor glycemic control, and in the satisfaction domain scores in individuals with good and poor control.

**Table 3:** Scores of the questionnaires DQOL-BR and DKN-A at V1 and V5

Variables	Glycemic control (HbA1c)	V1	V5	p value
DKN-A	Good	7.18 ± 3.27	8.62 ± 2.46	0.0296**
	Regular	8.70 ± 2.43	8.50 ± 3.52	0.8309**
	Poor	8.61 ± 3.59	9.55 ± 2.59	0.2791**
	Total	7.90 ± 3.15	8.86 ± 2.73	0.1409**
Total DQOL-BR	Good	2.10 ± 0.45	2.01 ± 0.47	0.4139*
	Regular	2.09 ± 0.73	1.99 ± 0.52	0.5693*
	Poor	2.55 ± 0.51	2.09 ± 0.43	0.0033*
	Total	2.60 (2.1, 3.3)	2.20 (1.87, 2.43)	0.0003*
DQOL-BR satisfaction	Good	2.46 (2.27, 2.73)	2.2 (2.2, 2.27)	0.0382*
	Regular	2.2 (1.91, 2.67)	2.08 (1.63, 2.55)	0.3668*
	Poor	3.26 (2.47, 3.56)	2.4 (2.12, 2.67)	0.0249*
	Total	2.11 (1.75, 2.11)	2.1 (1.67, 2.3)	0.1656*
DQOL-BR impact	Good	1.85 (1.72, 1.89)	2.08 (1.51, 2.2)	0.9000*
	Regular	1.77 (1.51, 2.48)	2.17 (1.65, 2.44)	0.9687*
	Poor	2.58 (1.89, 2.80)	2.10 (1.83, 2.41)	0.0201*
	Total	1 (1, 1.57)	1.13 (1, 1.7)	0.4698*
DQOL-BR Social/Vocational concerns	Good	1.14 (1.00, 1.57)	1.13 (1, 2.15)	0.3323*
	Regular	1.00 (1.00, 1.03)	1.05 (1, 1.61)	0.0519*
	Poor	1.21 (1.00, 1.81)	1.12 (1, 1.7)	0.8433*
	Total	2.25 (1.75, 2.87)	1.75 (1.5, 2.37)	0.0459*
DQOL-BR concerns related to the DM	Good	2.00 (1.31, 2.62)	1.87 (1.50, 2.25)	0.2829*
	Regular	2.00 (1.50, 2.12)	1.75 (1.25, 2.25)	0.9044*
	Poor	2.37 (2.00, 2.81)	2.25 (1.75, 2.50)	0.2551*
	Total	2.30 ± 0.57	1.98 ± 0.44	0.0035*

* Paired Wilcoxon test

** Paired *t* test

## Discussion

The sociodemographic characteristics of the patients evaluated in this study are compatible with those expected for age- and gender-matched patients with T2DM. However, it is important to emphasize that the study cohort had an important characteristic: the fact that it had a single modifiable risk factor—lack of physical activity—since most individuals reported not smoking nor abusing alcoholic beverages. This information is important for health professionals who need to find means to stimulate individuals with DM to practice physical activity.

Low family income, in turn, limits the implementation of appropriate treatment because it interferes with the choices of food and therapeutic plan, as discussed below.

As for the clinical characteristics, the mean values of BMI, WC, and WC/HC ratio associated with the increased prevalence of hypertension, obesity, and dyslipidemia, reflect the high frequency of diagnostic criteria for metabolic syndrome, as expected for the sample of subjects in the study. A significant reduction in BMI and WC values in some groups of patients observed in this study suggest an improvement in treatment adherence, with potential benefits to the glycemic control.

The high proportion of individuals classified as having a high or medium risk for DM complications, associated with the fact that most of these individuals had poor glycemic control, shows greater dependence from health professionals and higher treatment costs, more specifically with hospitalization, diagnostic tests, and treatment of complications. These figures reflect the current Brazilian situation, where health expenditures related to DM in 2015 were estimated at 21 billion US Dollars [2]. We should emphasize that hospitalizations commonly reflect an inadequate DM control. In this sense, a study carried out in the state of Paraná showed an increasing tendency of hospitalization due to DM in men aged 50–59 years and in those older than 80 years [34].

The fact that most patients managed their DM with oral antidiabetic agents alone is within the expected for the average disease duration observed in our study (9 years), which corresponds to a disease phase in which the individual still has a residual pancreatic secretory function [6]. However, the fact that a sizeable portion of patients required modification of their prescribed pharmacological regimen reflects the high rates of poor glycemic control observed in other studies conducted in Brazil [8, 9]. The requirement for insulin therapy or adjustment in the insulin regimen may be pointing to inertia in medical therapy, because when the HbA1c values are maintained above 7 %, even with maximum doses of two oral antidiabetic agents, there is an increased risk of cardiovascular complications [35]. The use of new classes of oral antidiabetic agents could probably help decrease the rates of poor glycemic control, but the high cost of these drugs makes their acquisition difficult by most patients receiving care at the public health system in Brazil, a fact that was confirmed in this study, since most patients had income between two and five minimum wages and only four patients had resources to change the class of the oral antidiabetic drug before starting an insulin regimen.

Among the participants of this study, the main risk factor for complications was poor glycemic control, which was the result of inadequate prescribed and/or implemented pharmacological treatment, since more than half of the patients required treatment adjustment, and non-adherence to non-pharmacological treatment guidelines, including lifestyle habits such as nutrition, practice of physical activity, alcohol consumption, and smoking. Health professionals may have a strong influence on how patients with DM perceive their disease and follow treatment recommendations. The patients, in turn, need to find means to make their actions result in effective lifestyle changes, and in a more forceful way, in weight control and loss in individuals with T2DM. Patients’ motivation and attitude fluctuate during treatment and are influenced by cognitive, motivational, and emotional factors, and may be stimulated by health professionals [36].

The benefits of group education for individuals with T2DM have been previously demonstrated [11, 12]. A study [37] has shown that education of individuals with T2DM with the CMs instrument [33] was more effective in groups than individually, with a 1.4 % reduction in HbA1c in the intervention group and 0.5 % in the control group, in addition to improvements in lipid profiles in both groups.

Despite our observations about improved DM learning in the group with good glycemic control and an increased percentage of individuals with scores ≥8, the ability of individuals with T2DM to understand and retain information provided by health professionals in primary care [7] is still relatively low. In our evaluation of the level of satisfaction associated with DM, the scores were similar to those in a study carried out in Curitiba (Paraná) [32], which characterized a dissatisfaction with the disease. The comparison of the satisfaction level before and after the intervention showed a partial improvement in the total score and in the satisfaction and impact domains, but still hints a dissatisfaction with the DM.

Although the present study was unable to establish on an individual basis the effect of the pharmacological adjustment or the application of the CMs, the fact that the percentage of individuals with scores ≥8.0 in the DKN-A increased after the intervention from 51.9 to 65.4 % suggests an improvement in the participants’ knowledge about the disease after application of the CMs. However, it is important to reinforce that knowledge alone may not be sufficient to change health behaviors, especially among men and older individuals (since studies have shown that women are more prone to behavioral changes) [38, 39]. Also, the use of messages and content more appropriate to the cultural standard of these individuals is recommended to promote changes in daily behaviors. In this regard, health professionals need to evaluate in their practices the degree of knowledge that the patients have about their disease, and the impact that the disease has on their lives, enabling a therapeutic plan more congruent with the condition of each patient.

In this study, the mean HbA1c at V1 was 8.91, and 67.3 % of the subjects presented an inadequate glycemic control. These data are close to those of a large Brazilian multicenter study [8] in which 73 % of the patients with T2DM had an inadequate glycemic control (HbA1c >7 %). Another study carried out in Curitiba (Paraná) [40] had a quite low proportion of patients with T1DM and T2DM who reached all targets of good control recommended by the Brazilian Society of Diabetes regarding HbA1c levels, BP, BMI, and lipids, and the median HbA1c levels were 9.0 and 7.8 % in patients with T1DM and T2DM, respectively. Although this situation seems to be better in the U.S., an American study [10] has demonstrated that 47.8 % of the individuals with DM had an HbA1c level above 7 %. In Germany, a study [41] adopting an HbA1c below 6.5 % as a criterion of good control, found that 52.7 % of 5135 individuals with T2DM had an inadequate control. These data show a need, even in developed countries, for more effective measures to improve glycemic control and reduce complications associated with DM. In the present study, we observed a 0.69 % reduction in mean HbA1c from V1 to V4 (p = 0.0003) and 0.46 % from V1 to V5 (p = 0.0219). It is unclear why the glycemic control worsened from V4 to V5 (mean HbA1c increased in 0.24 % between these time points, p = 0.03), but it may have been influenced by intercurrent events during this period in some individuals, such as hospital admissions, infections, emotional factors, or even a reduction in adherence to non-pharmacological treatment, such as diet errors and/or decrease in physical activity. The reduction in HbA1c level obtained with the intervention was more evident when we considered the group of 35 patients (67.3 %) with inadequate glycemic control at two moments (3 months, p = 0.0001; and 6 months, p = 0.0136). The same trend of improvement in glycemic control after the intervention was observed in individuals requiring an adjustment in pharmacological treatment (63.5 %), who presented a 0.6 % reduction in mean HbA1c during 6 months of follow-up.

Education in DM is one of the most important factors to decrease the current high rates of poor glycemic control, particularly in Brazil. As already demonstrated in previous studies [10, 11, 37], cost-effectiveness is more favorable in adults with T2DM with educational programs conducted in groups, whereas in T1DM patients, improvements are more noticeable in psychosocial aspects [13]. The educational level certainly influences an individual’s capacity to grasp the content offered in educational programs. Even with the city of Maringá occupying the 23rd position on the Municipal Human Development Index (*Índice de Desenvolvimento Humano Municipal,* IDHM 2010) in Brazil [42], 59.6 % of the participants in this study did not complete primary school, and only 9.6 % completed a higher education program.

A patient’s knowledge about his or her disease is one of the pillars to develop self-care actions. However, simple knowledge acquisition may not necessarily translate into behavior and lifestyle changes, but may enhance the capacity and confidence of the patient to develop self-care actions, contributing to improve the management and prevention of chronic diseases such as DM.

The limitations of this study include the number of participants, which is small considering all individuals with DM receiving primary health care in that municipality. In any event, the rate of follow-up loss after study enrollment was relatively low (14.8 %), which indicates that the individuals who took part in the study valued the proposed activities.

We should also mention as other limitations some factors inherent to this type of study, such as the duration of the intervention, since 6 months may not have been sufficient for the participants to translate the information into learned knowledge, and a limited ability to generalize the results of this type of study, so the estimates are only valid for a population with similar characteristics, although the sample was obtained at random. The fact that we adopted the Ames analyzer (Bayer Diagnostics, DCA 2000) as an instrument to evaluate the HbA1c level instead of the conventional HPLC method may also be a limitation. However, the choice to use the Ames analyzer emerged from a limitation of having the HbA1c tested at the public health system, which recommends that this test should only be performed at an interval of at least 6 months. We believe that this did not represent a problem in the interpretation of the results since there is a strong statistical correlation between the two methods, with a correlation coefficient of 0.95 [21], as observed in the present study (0.91).

Lastly, although the change of treatment regimen for some patients during the study may have represented a possible bias, this reinforces once again the need for a more effective follow-up of T2DM patients in primary care, including periodic evaluation of a specialist.

Despite these limitations, the results of this study indicate a significant (p < 0.05) mean increase of 1.4 points in DM knowledge in patients with good glycemic control, and a decrease in mean HbA1c levels, BMI, and WC in some individuals. This signals the perspective of the actions of health professionals in reaching this type of population.

## Conclusion

This study demonstrated an inadequate degree of knowledge and satisfaction related to DM, and a high frequency of T2DM patients with inadequate glycemic control in primary care. The intervention of a mobile consultancy specialized in DM adjusted the pharmacological treatment of 63.5 % of the patients and obtained partial improvement in blood glucose control, degree of knowledge about DM, and satisfaction related to the disease, in addition to reducing the BMI and WC in some individuals. These findings indicate a need for more effective care and educational measures to decrease the complications and mortality related to the disease.
